# Reproducibility of Ki67 Haralick entropy as a prognostic marker in estrogen receptor–positive HER2-negative breast cancer

**DOI:** 10.1093/ajcp/aqaf081

**Published:** 2025-08-09

**Authors:** Dovile Zilenaite-Petrulaitiene, Allan Rasmusson, Ruta Barbora Valkiuniene, Aida Laurinaviciene, Linas Petkevicius, Arvydas Laurinavicius

**Affiliations:** Center for Digital Medicine, Translational Health Research Institute, Faculty of Medicine, Vilnius University, Vilnius, Lithuania; Department of Pathology and Forensic Medicine, Institute of Biomedical Sciences, Faculty of Medicine, Vilnius University, Vilnius, Lithuania; National Center of Pathology, affiliate of Vilnius University Hospital Santaros Klinikos, Vilnius, Lithuania; Institute of Computer Science, Faculty of Mathematics and Informatics, Vilnius University, Vilnius, Lithuania; Center for Digital Medicine, Translational Health Research Institute, Faculty of Medicine, Vilnius University, Vilnius, Lithuania; Department of Pathology and Forensic Medicine, Institute of Biomedical Sciences, Faculty of Medicine, Vilnius University, Vilnius, Lithuania; National Center of Pathology, affiliate of Vilnius University Hospital Santaros Klinikos, Vilnius, Lithuania; Department of Pathology and Forensic Medicine, Institute of Biomedical Sciences, Faculty of Medicine, Vilnius University, Vilnius, Lithuania; National Center of Pathology, affiliate of Vilnius University Hospital Santaros Klinikos, Vilnius, Lithuania; Center for Digital Medicine, Translational Health Research Institute, Faculty of Medicine, Vilnius University, Vilnius, Lithuania; Department of Pathology and Forensic Medicine, Institute of Biomedical Sciences, Faculty of Medicine, Vilnius University, Vilnius, Lithuania; National Center of Pathology, affiliate of Vilnius University Hospital Santaros Klinikos, Vilnius, Lithuania; Institute of Computer Science, Faculty of Mathematics and Informatics, Vilnius University, Vilnius, Lithuania; Center for Digital Medicine, Translational Health Research Institute, Faculty of Medicine, Vilnius University, Vilnius, Lithuania; Department of Pathology and Forensic Medicine, Institute of Biomedical Sciences, Faculty of Medicine, Vilnius University, Vilnius, Lithuania; National Center of Pathology, affiliate of Vilnius University Hospital Santaros Klinikos, Vilnius, Lithuania

**Keywords:** Ki67 proliferation rate, Haralick texture entropy, breast cancer, digital image analysis, spatial heterogeneity, immunohistochemistry, prognostic biomarker

## Abstract

**Objective:**

Intratumoral heterogeneity (ITH) of Ki67 expression reflects the proliferative diversity of breast cancer (BC) cells and has been associated with disease progression. Quantification of Ki67 ITH using Haralick entropy metric from digital image analysis (DIA) has been reported as an independent predictor of breast cancer–specific survival (BCSS); however, its reproducibility across DIA platforms and dependence on tumor tissue sampling have not been investigated.

**Methods:**

Whole-slide images of Ki67-stained tumor sections from 254 patients with ER+/HER2− BC were analyzed independently using HALO and Aiforia DIA platforms. The DIA outputs were subsampled using hexagonal grids to compute Ki67 Haralick entropy. Reproducibility was tested across DIA platforms and under simulated surgical excision and core biopsy scenarios. Lastly, the impact on prognostic modeling for BCSS was assessed.

**Results:**

Haralick entropy demonstrated strong Ki67 ITH cross-platform reproducibility. For prognosis, it provided stronger model performance than conventional Ki67% metrics and independently predicted worse BCSS alongside lymph node involvement. Its prognostic value remained consistent across simulated sampling scenarios.

**Conclusions:**

Ki67 Haralick entropy is a reproducible and robust image-derived ITH metric in ER+/HER2− BC. It demonstrated improved prognostic modeling performance compared to conventional Ki67% across 2 different DIA platforms and sampling conditions, supporting its potential for clinical implementation.

**KEY POINTS**

- Limited reproducibility of proliferative properties of breast cancer assessed by Ki67 immunohistochemistry hinders its clinical adoption. A computational biomarker, Ki67 Haralick entropy, has been reported to improve prognostic stratification of patients.
- Reproducibility assessment across 2 digital image analysis platforms and sampling conditions reveals Ki67 Haralick entropy as a robust prognostic biomarker in patients with ER+/HER2− breast cancer.
- Haralick entropy of Ki67 expression provides independent prognostic value and demonstrates stronger model performance than Ki67%, supporting its potential clinical relevance.

## INTRODUCTION

Intratumoral heterogeneity (ITH) reflects the diverse cellular composition within tumors, encompassing genetic, phenotypic, and functional variations that drive tumor progression, treatment resistance, and relapse.^1^ These variations enable resistant subpopulations to evade treatment, driving metastasis and recurrence.^2^ In breast cancer (BC), ITH has been associated with poor survival outcomes and reduced treatment efficacy,^3^ highlighting the need for robust ITH assessment to advance prognostic predictions and support personalized treatment strategies.

Intratumoral heterogeneity is commonly evaluated using molecular and/or image-based techniques, including advanced spatial transcriptomic, proteomic, and RNA imaging technologies.^4-7^ While molecular methods often lack spatial context, spatial biology image-based approaches suffer from capacity and cost constraints, particularly when analyzing full-face tissue microscopy sections. Digital image analysis (DIA) of whole-slide images (WSIs), with advanced machine learning algorithms, addresses these limitations by enabling high-throughput analysis of large pathology data sets.^8,9^ This approach precisely and accurately quantifies immunohistochemistry (IHC) biomarkers such as Ki67, a widely used proliferation marker.^10,11^ In contrast to routine visual scoring, which typically relies on global estimates of the whole tumor section or hotspot evaluations by pathologists, DIA enables reproducible assessment of Ki67 expression across tumor regions. Besides basic quantifications, DIA also enables spatially resolved insights into biomarker distributions within the tumor microenvironment.^10,12-14^

Among DIA methodologies, texture-based spatial entropy metrics adapted from ecological diversity studies have emerged as promising features for quantifying ITH.^15^ Indicators such as Shannon entropy, which captures both the richness and evenness of distribution, and Simpson’s index, which reflects the probability that 2 randomly selected entities belong to the same category and emphasizes dominant components, have demonstrated clinical relevance in BC. The Simpson index was shown to reflect the ITH of estrogen receptor (ER) and predicted patient prognosis,^16^ while Shannon entropy was associated with shorter BC-specific survival (BCSS) and disease-free survival in early-stage ER-positive and human epidermal growth factor 2 (HER2)–negative (ER+/HER2–) BC.^10^

Grid-based spatial texture analysis further enhances ITH evaluation by quantifying regional variability of biomarker expression. Haralick texture features, such as entropy, homogeneity, contrast, dissimilarity, and energy, were originally developed to describe variations in pixel intensities across digital images. However, when applied to grid-based subsampling of DIA data, these features enable quantification of ITH across WSIs.^17^ Plancoulaine et al^18^ and Laurinavičius et al^19^ demonstrated the prognostic value of Haralick texture and Ashman D bimodality indicators in BC. Similarly, Zilenaite et al^12^ reported that Ki67 and progesterone receptor (PR) ITH independently predicted overall survival in hormone receptor–positive BC. In a study of 254 ER+/HER2– BC cases, Haralick texture (homogeneity, entropy, contrast, dissimilarity, energy) and Ashman’s D indicators were computed for Ki67, ER, and PR using systematic hexagonal grid subsampling of DIA data.^14^ Among them, Ki67 Haralick texture entropy, which quantifies variability in Ki67 expression across grid elements, serves as an indicator of spatial heterogeneity of biomarker expression. Along with lymph node involvement, Ki67 spatial heterogeneity was identified as an independent adverse prognostic feature for BCSS, showing greater prognostic value than global Ki67% and other ITH indicators. Furthermore, when integrated with immune-related features, Ki67 entropy was shown to provide additional prognostic information,^20^ highlighting the added value of ITH assessment in prognostic modeling.

Despite the evidence accumulated, translation of Ki67 Haralick entropy into clinical practice first requires validation of its technical robustness. Specifically, its reproducibility across different DIA platforms and sensitivity to tumor tissue sampling conditions remain underinvestigated. Factors such as tissue segmentation and cell detection algorithms, as well as differences in tiling strategies and sampling methods (eg, surgical excision vs core biopsy), can affect ITH scores.^21-23^ While Failmezger et al^24^ demonstrated consistent ITH scores across tile sizes and grid positions, further validation across different DIA platforms and tumor sampling scenarios is essential to promote clinical applicability.

This study investigates the reproducibility and prognostic value of Ki67 ITH, quantified by Haralick texture entropy, in ER+/HER2– BC. Utilizing independent DIA outputs from the HALO and Aiforia platforms, we assessed the consistency of ITH metrics. Furthermore, we evaluated tumor tissue sampling scenarios by simulating variable surgical excision sample sizes and core biopsy shapes to evaluate their impact on ITH metrics and the prognostic models.

## METHODS AND MATERIALS

### Patients and tumor tissue specimens

This study included 254 patients with surgically resected primary invasive (stages I-III) ER+/HER2– BC treated at the National Cancer Institute (Vilnius, Lithuania) between 2007 and 2014, as described previously.^14^ Pathology diagnoses were performed at the National Center of Pathology, an affiliate of Vilnius University Hospital Santaros Klinikos (Vilnius, Lithuania). Of an initial cohort of 264 patients, 10 were excluded due to receiving neoadjuvant therapy, presenting with distant metastases at diagnosis, lacking comprehensive clinicopathologic data, or being younger than 35 years. Clinical data were collected from medical records, including age, stage, histologic grade, tumor size, lymph node status, and intrinsic BC subtype. Breast cancer–specific survival was defined as the time from the date of surgery to BC-related death, with follow-up censored at 10 years postsurgery.

Ethical approval for the study was granted by the Lithuanian Bioethics Committee (reference: 40, 03/08/2007, number: 33). Written informed consent was obtained from all patients. For ITH analysis on anonymized samples, the requirement for individual consent was waived (approval updates: 12/09/2017, 6B-17-189; 12/01/2023, 6B-23-8).

### Sample preparation, IHC staining, and visual Ki67 assessment

**Figure 1:**
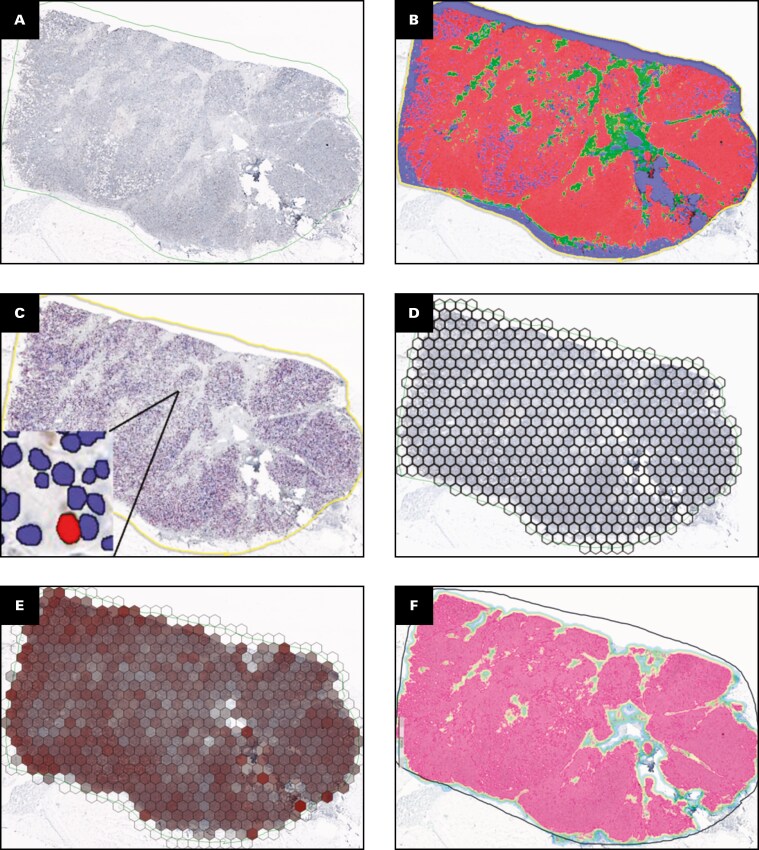

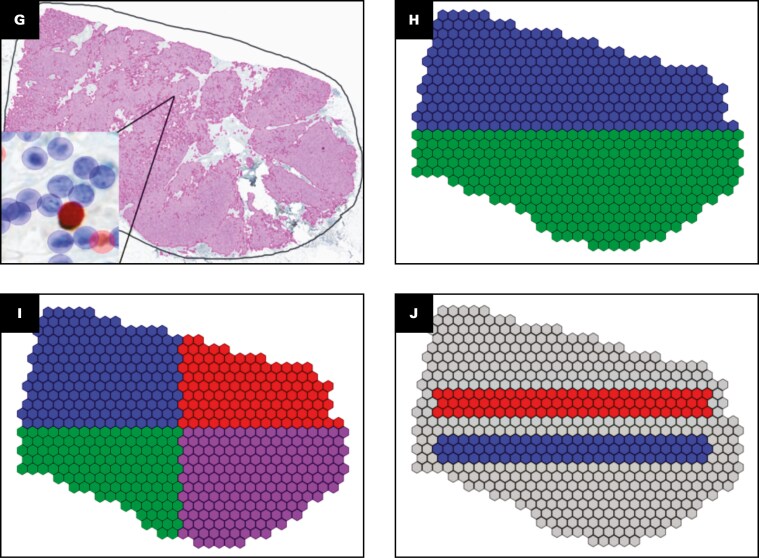
Study workflow and example of Ki67 immunohistochemistry (IHC) analysis by digital image analysis (DIA) algorithms. **A**, Whole-slide image of breast cancer tissue immunohistochemically stained for Ki67, scanned at 20× magnification to capture high-resolution detail. **B**, Pixelwise segmentation of tumor epithelium and stroma using the HALO AI classifier. Tumor regions are depicted in red, stroma in green, and background in blue. **C**, HALO Multiplex IHC quantitative DIA of Ki67 staining, focusing on the entire tumor tissue region outlined in yellow. Ki67-positive cells are highlighted in red, while Ki67-negative cells are shown in blue. **D**, Hexagonal grid overlay (hexagon side length: 262.5 µm, equivalent to 1050 pixels) applied to systematically subsample the Ki67 digital analysis results. **E**, Visualization of Ki67-positive cell percentages within each hexagon from subsampling in **D**—transparent hexagons have 0 or low positivity while darker red shades show higher positivity. Visually, it shows the spatial heterogeneity later captured by the entropy measures. **F**, Pixelwise segmentation of tumor epithelium and stroma using Aiforia AI, focusing on the tumor tissue region outlined in black. Tumor regions are indicated in pink. **G**, Aiforia AI quantitative DIA of Ki67 IHC. Ki67-positive cells are marked in red and Ki67-negative cells in blue. **H**, Tumor tissue subsampled into top (blue) and bottom (green) halves for reproducibility analysis of Ki67% and intratumoral heterogeneity (ITH) indicators using HALO DIA data. **I**, Tumor tissue subsampled into quadrants for reproducibility analysis of Ki67% and ITH indicators, with the northwest quadrant highlighted in blue, northeast in red, southeast in purple, and southwest in green. **J**, Simulated core biopsy extraction from the tumor slide, approximating real biopsy dimensions (1.2 mm width by 12 mm length) for reproducibility assessment of Ki67% and ITH indicators. Biopsy 1 is shown in red and biopsy 2 in blue.

Formalin-fixed, paraffin-embedded tissue blocks with the highest invasive tumor content were selected for the study. One 3-µm-thick section per case was stained for Ki67 with the MIB-1 antibody (1:200 dilution; Dako) Figure 1A. Immunohistochemistry was performed using the Roche Ventana BenchMark ULTRA automated stainer (Ventana Medical Systems). The detailed staining protocol is available at http://dx.doi.org/10.17504/protocols.io.q26g71qxqgwz/v1.

Visual Ki67% assessment was performed in accordance with routine diagnostic pathology practice, following the recommendations of the International Ki67 in Breast Cancer Working Group.^25^ For each case, Ki67% was visually estimated by examining the entire invasive tumor area and determining the proportion of positively stained tumor nuclei. The estimate was based on the visual assessment of at least 500 invasive tumor cells across multiple representative regions, without restricting the evaluation to hotspot areas. All assessments were performed on the same WSIs that were subsequently used for DIA.

### DIA and ITH indicators calculations

The DIA process for calculating Haralick entropy of Ki67-stained WSIs using the HALO platform (Indica Labs) has been described previously.^14,20^ Briefly, Ki67-stained slides were scanned using an Aperio AT2 DX Slide Scanner (Leica Aperio Technologies) at 20×, with a pixel size of 0.5003 µm. A subset of 15 WSIs was randomly selected to represent a range of tumor morphologies and staining patterns. Tumor, stroma, and background regions (including necrosis, artifacts, and glass) were manually annotated by a pathologist and used to train, validate, and test a HALO AI DenseNet model (v3.5.3577; Figure 1B) for automated tissue classification. The data set was split into training (n = 9), validation (n = 3), and test (n = 3) subsets. Model performance, assessed using the HALO AI validation tool, yielded *F* scores of 0.93 for tumor, 0.90 for stroma, and 0.99 for glass/background detection on the test set. Following tissue classification, all tumor cell nuclei were segmented using the HALO Multiplex IHC algorithm, and each nucleus was classified as Ki67-positive or Ki67-negative based on nuclear staining intensity Figure 1C. Nontumor tissue was excluded from the analysis. Global Ki67% values were derived per case by calculating the proportion of Ki67-positive tumor cells among all tumor cells identified in the DIA output. For spatial heterogeneity analysis, cell coordinates were extracted for each WSI to enable spatial tile-based analysis. A hexagonal grid with side lengths of 262.5 µm (1050 pixels) and an area of approximately 178 590 µm² per tile was overlaid on the region of interest Figure 1D. Hexagons with fewer than 50 cells were excluded from further analysis because they were considered insufficiently sampled. Within each hexagon, the percentage of Ki67-positive cells was calculated Figure 1E and ranked into 10 intervals (0%-10%, >10%-20%, etc) to generate a co-occurrence matrix. Haralick features were then calculated, focusing on entropy, which has demonstrated independent prognostic value in this BC cohort.^14^

To assess reproducibility, the same WSIs used for HALO analysis were provided in a blinded manner for independent DIA using the Aiforia AI platform (Aiforia Technologies) Figure 1F and G. The analysis was performed using the platform’s standard pretrained algorithm without additional fine-tuning or retraining. Tumor and stromal regions were automatically segmented using Aiforia’s built-in tissue classification tool. Subsequently, all cell nuclei within the tumor regions were segmented and classified as Ki67-positive or Ki67-negative based on nuclear staining intensity. Global Ki67% was calculated per case as the proportion of Ki67-positive tumor cells among all tumor cells identified in the Aiforia DIA output. For spatial analysis, the same hexagonal grid-based pipeline as in HALO was applied to compute Haralick entropy of Ki67 expression. Further, HALO DIA outputs were used to assess the dependence of these metrics under different tissue sampling conditions. The tumor tissue was subsampled into smaller but still connected sections by using halves and quadrants (Figure 1H and I, respectively), and core biopsy samples, approximating real biopsy dimensions (1.2 mm width, 12 mm length), were generated vertically, assuming a random tissue orientation Figure 1J.

### Statistical analysis and prognostic modeling

Statistical analyses were performed using SAS (v9.4; SAS Institute) and R (v4.1.0; R Project for Statistical Computing). All tests were 2-sided, with significance set at *P* < .05. The normality of continuous variables was tested using the Kolmogorov-Smirnov test. All Ki67% values from visual assessment and DIA (HALO, Aiforia), as well as Haralick entropy values, showed significant deviation from normality (*P* < .01), even after logarithmic transformation; therefore, nonparametric methods were applied in univariate comparisons and correlation analyses. The correlation between Ki67% and Haralick entropy estimates was analyzed using Spearman rank correlation coefficients. Agreement was further assessed using intraclass correlation coefficients (ICCs), concordance correlation coefficients (CCCs), and Bland-Altman plots for visual evaluation. The ICCs were computed using a 2-way mixed-effects model for single-rater consistency [ICC(3,1)], as implemented in the psych R package. Given the nonnormal distribution of the input data, ICC values were interpreted with caution, following prior recommendations for large-sample reproducibility studies.^26,27^ The CCCs were calculated using Lin’s method, with 95% CIs estimated by nonparametric bootstrap resampling (1000 replicates). Wilcoxon signed-rank tests were employed to evaluate reproducibility under different sampling conditions. Cutoff values were identified separately for each variable using the publicly available Cutoff Finder tool (Charité University), applying the log-rank test method to determine the threshold that resulted in the most significant separation of BCSS.^28^

Univariate Cox regression was performed to assess the prognostic value of clinicopathologic, Ki67%, and ITH indicators. Results are presented as hazard ratios (HRs) with 95% CIs and corresponding *P* values. Kaplan-Meier plots were used to estimate BCSS distributions, with differences in survival times assessed using the log-rank test. Variables showing *P* < .05 in univariate analysis were incorporated in multivariable Cox proportional hazards models. In all models, lymph node status and tumor grade were included as core clinicopathologic covariates, while different Ki67% and ITH indicators (including regional or simulated biopsy-derived measures) were incorporated depending on the specific analytic comparison. Models were fitted using the CoxBoost R package, which applies likelihood-based boosting for variable selection. The optimal penalty parameter was identified using the optimCoxBoostPenalty function, and the number of boosting steps was optimized via 5-fold cross-validation using cv.CoxBoost. The data set was split into training (190 patients) and testing (64 patients) subsets. Model performance was assessed using Harrell’s C-index and likelihood ratio (LR) tests to determine BCSS prediction accuracy.

## RESULTS

### Summary statistics of clinicopathologic, Ki67%, and ITH indicators

This study included 254 female patients with BC with a median age of 62 years and a median follow-up of 114.8 months (IQR, 109.2-120.0 months). During the 10-year follow-up, 34 (13.4%) BC-related deaths occurred. Most tumors were T1 (57.5%), histologic grade 2 (59.1%), and lymph node negative (59.1%). The cohort comprised 48.0% luminal A–like and 52.0% luminal B–like (HER2−) subtypes. Clinicopathologic characteristics of all patients, including stratification by BCSS status, are presented in Table S1.

Summary statistics for Ki67% and ITH indicators are presented in Table 1. Pathologist-reported Ki67% was evaluated on the entire tumor tissue area, with values ranging from 3.00% to 95.00%, reported as whole percentages. In contrast, Ki67% values derived from the HALO and Aiforia DIA platforms ranged from 0.39% to 85.68% for HALO and 0.09% to 87.67% for Aiforia, and they were reported with 2 decimal places for clarity. Pathologist-reported Ki67% had a median of 20% (IQR, 10.00%-30.00%), which was higher than medians from DIA platforms: HALO (12.42%; IQR, 5.98%-23.20%) and Aiforia (10.77%; IQR, 5.00%-20.78%). Haralick entropy ranged from 0.00 to 5.13 for HALO and 0.00 to 5.06 for Aiforia. HALO-based Haralick entropy medians were 2.38 (IQR, 1.03-3.31), exceeding Aiforia-based medians of 2.02 (IQR, 0.66-3.06), although the difference was not statistically significant (*P* = .1181). Tumor subsamples and simulated biopsy specimens analyzed using HALO revealed consistent Ki67% medians across halves (11.89%-12.57%), quadrants (11.25%-12.33%), and biopsy specimens (11.70%-12.20%). Similarly, Haralick entropy medians ranged from 2.07 to 2.35 across subsamples and core biopsy specimens, indicating stable and reproducible ITH metrics.

**Table 1:** Summary Statistics of Ki67% and Intratumoral Heterogeneity Indicators

Indicator	Mean (sd)	Median	IQR	Minimum	Maximum	*P*-value
Ki67% (Visual)	21.96 (15.26)	20.00	10.00-30.00	3.00	95.00	<.001^a^
Ki67% (HALO)	16.53 (15.00)	12.42	5.98-23.20	0.39	85.68	.07
Ki67% (Aiforia)	13.97 (12.56)	10.77	5.00-20.78	0.09	87.67	
Ki67% (HALO, top half)	16.46 (15.02)	11.89	5.82-22.82	0.31	85.24	.71
Ki67% (HALO, bottom half)	16.36 (14.72)	12.57	5.96-21.68	0.30	85.06	
Ki67% (HALO, southeast quadrant)	16.27 (15.00)	11.25	5.73-22.06	0.00	89.17	>.05^b^
Ki67% (HALO, northeast quadrant)	16.33 (14.84)	12.18	5.83-22.70	0.09	90.21	
Ki67% (HALO, southwest quadrant)	16.51 (15.04)	12.24	5.90-22.64	0.35	83.29	
Ki67% (HALO, northwest quadrant)	16.36 (14.97)	12.33	5.96-21.91	0.37	82.83	
Ki67% (HALO, biopsy 1)	16.17 (14.79)	11.70	5.95-21.86	0.27	81.77	.07
Ki67% (HALO, biopsy 2)	16.36 (15.25)	12.20	5.87-22.67	0.00	80.94	
Ki67 Haralick entropy (HALO)	2.24 (1.40)	2.38	1.03-3.31	0.00	5.13	.12
Ki67 Haralick entropy (Aiforia)	1.98 (1.39)	2.02	0.66-3.06	0.00	5.06	
Ki67 Haralick entropy (HALO, top half)	2.16 (1.39)	2.27	0.99-3.26	0.00	5.02	.34
Ki67 Haralick entropy (HALO, bottom half)	2.20 (1.38)	2.35	0.99-3.29	0.00	5.06	
Ki67 Haralick entropy (HALO, southeast quadrant)	2.08 (1.37)	2.18	0.78-3.14	0.00	5.07	>.05^c^
Ki67 Haralick entropy (HALO, northeast quadrant)	2.09 (1.37)	2.22	0.89-3.21	0.00	5.27	
Ki67 Haralick entropy (HALO, southwest quadrant)	2.07 (1.37)	2.23	0.95-3.13	0.00	5.19	
Ki67 Haralick entropy (HALO, northwest quadrant)	2.07 (1.33)	2.13	0.89-3.18	0.00	4.71	
Ki67 Haralick entropy (HALO, biopsy 1)	2.01 (1.35)	2.14	0.72-3.04	0.00	4.64	.82
Ki67 Haralick entropy (HALO, biopsy 2)	1.98 (1.34)	2.07	0.65-3.10	0.00	4.64	

^a^Wilcoxon signed-rank test comparing visual Ki67% estimates with digital image analysis–derived values (HALO and Aiforia) yielded *P* < .001. ^b^Wilcoxon signed-rank tests for paired comparisons of Ki67% across tumor subsampling regions showed no significant differences: southeast vs northeast (*P* = .96), southeast vs southwest (*P* = .44), southeast vs northwest (*P* = .65), northeast vs southwest (*P* = .86), northeast vs northwest (*P* = .57), and southwest vs northwest (*P* = .48). ^c^Wilcoxon signed-rank tests for paired comparisons of Ki67 Haralick entropy across tumor subsampling regions also showed no significant differences: southeast vs northeast (*P* = .90), southeast vs southwest (*P* = .71), southeast vs northwest (*P* = .60), northeast vs southwest (*P* = .47), northeast vs northwest (*P* = .59), and southwest vs northwest (*P* = .86).

### Agreement of Ki67 indicators between DIA platforms

**Figure 2:**
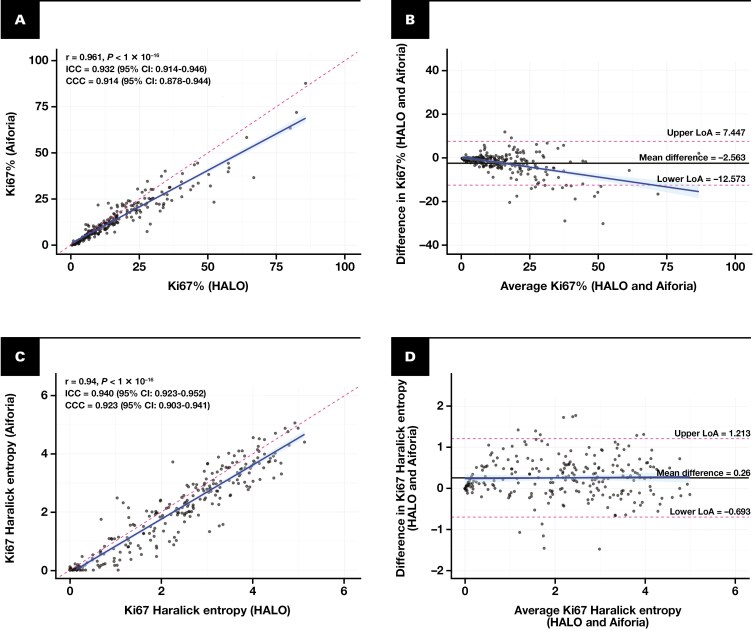
Comparisons of Ki67% and Haralick entropy estimates from 2 digital image analysis (DIA) platforms: HALO and Aiforia. **A**, **C**, Scatterplots for pairwise comparisons: (**A**) Ki67% (HALO) vs Ki67% (Aiforia) and (**C**) Haralick entropy (HALO) vs Haralick entropy (Aiforia). The dashed pink line represents the identity line (1:1), and the blue solid line shows the linear regression fit with a 95% CI displayed as light blue shading. Each plot includes Spearman correlation coefficient (*r*) with *P* value, intraclass correlation coefficient with 95% CI, and concordance correlation coefficient with 95% CI to quantify agreement between methods. **B**, **D**, Bland-Altman plots for the same comparisons: (**B**) Ki67% and (**D**) Haralick entropy. The solid black line represents the mean difference, while the dashed pink lines indicate the limits of agreement, defined as ±1.96 standard deviations from the mean difference. The blue solid line shows the linear regression of differences with a 95% CI (light blue shading).

Ki67% assessed by DIA was systematically lower than pathologist-reported values, possibly reflecting the tendency of pathologists to preferentially assess hotspots in certain cases (*P* < .0001; Figure S1). Pairwise comparisons revealed no significant differences in Ki67% or Haralick entropy between HALO and Aiforia (*P* > .05, Table 1). Both metrics demonstrated strong concordance, with Spearman correlation coefficients of 0.96 for Ki67% and 0.94 for Haralick entropy (*P* < .0001; Figure 2A and C, respectively). The ICCs were 0.93 (95% CI, 0.91-0.95) for Ki67% and 0.94 (95% CI, 0.92-0.95) for Haralick entropy. Bland-Altman analyses indicated mean differences (biases) of –2.56% for Ki67% and 0.26 for Haralick entropy, which were small relative to their respective ranges. Limits of agreement ranged from –12.57 to 7.45 for Ki67% Figure 2B and –0.69 to 1.21 for Haralick entropy Figure 2D, highlighting the robustness and reliability of these metrics across platforms.

### Ki67% and ITH indicators in tumor subsampling and core biopsy simulation experiments

HALO DIA demonstrated high consistency in Ki67% and Haralick entropy estimates across tumor subsamples and simulated biopsy specimens. Spearman correlation coefficients ranged from 0.94 to 0.98 for Ki67% and from 0.88 to 0.99 for entropy (*P* < .0001; Table S2). The ICCs and CCCs ranged from 0.945 to 0.988 (95% CI, 0.922-0.994) for Ki67% and 0.879 to 0.985 (95% CI, 0.840-0.989) for Haralick entropy. Bland-Altman analyses confirmed minimal differences and stable limits of agreement under varying conditions, with mean differences ranging from –0.36 to 0.15 and limits of agreement from –9.76 to 9.58 for Ki67%, as well as mean differences from –0.26 to 0.02 and limits of agreement from –1.28 to 1.31 for Haralick entropy (Table S2). Wilcoxon tests revealed no statistically significant differences for Ki67% or Haralick entropy between tumor subsamples and simulated core biopsy specimens Table 1.

### Prognostic value of clinicopathologic, Ki67%, and ITH indicators

Univariate regression analysis results on BCSS are summarized in Table 2. Grade 3 tumors and lymph node involvement were significantly associated with worse BCSS among clinicopathologic variables. Other factors, such as patient age, stage, tumor invasion stage, and BC subtype, did not reveal univariate associations with BCSS in this cohort^14^ and were therefore excluded as stratification variables in further analyses.

**Table 2:** Univariate Cox Regression Analysis of Clinicopathologic, Ki67%, and Intratumoral Heterogeneity Indicators for Breast Cancer–Specific Survival

Univariate Cox regression analysis			
Indicator	Hazard ratio	*P* value	95% CI
Grade (G1-2 vs G3)	2.66	.004	1.36-5.22
Lymph node status (pN0 vs pN1-3)	2.29	.017	1.16-4.54
Ki67% (Visual)	3.01	.017	1.17-7.78
Ki67% (HALO)	2.31	.013	1.17-4.58
Ki67% (Aiforia)	2.29	.013	1.17-4.48
Ki67% (HALO, top half)	4.16	.033	1.00-17.38
Ki67% (HALO, bottom half)	2.15	.023	1.10-4.21
Ki67% (HALO, southeast quadrant)	2.15	.023	1.10-4.21
Ki67% (HALO, northeast quadrant)	2.58	.009	1.23-5.39
Ki67% (HALO, southwest quadrant)	3.91	.015	1.19-12.78
Ki67% (HALO, northwest quadrant)	2.37	.010	1.20-4.67
Ki67% (HALO, biopsy 1)	2.16	.023	1.10-4.25
Ki67% (HALO, biopsy 2)	2.05	.033	1.04-4.01
Ki67 Haralick entropy (HALO)	2.67	.003	1.36-5.26
Ki67 Haralick entropy (Aiforia)	2.71	.003	1.37-5.37
Ki67 Haralick entropy (HALO, top half)	2.27	.015	1.15-4.47
Ki67 Haralick entropy (HALO, bottom half)	2.30	.013	1.17-4.51
Ki67 Haralick entropy (HALO, southeast quadrant)	2.44	.007	1.24-4.78
Ki67 Haralick entropy (HALO, northeast quadrant)	2.75	.003	1.36-5.56
Ki67 Haralick entropy (HALO, southwest quadrant)	2.38	.010	1.21-4.69
Ki67 Haralick entropy (HALO, northwest quadrant)	2.59	.007	1.26-5.32
Ki67 Haralick entropy (HALO, biopsy 1)	2.26	.016	1.14-4.47
Ki67 Haralick entropy (HALO, biopsy 2)	3.10	.008	1.28-7.48

Ki67% indicators were significant predictors of worse BCSS, with HRs of 3.01 (95% CI, 1.17-7.78; *P* = .0170) for visual assessment, 2.31 (95% CI, 1.17-4.58; *P* = .0131) for HALO-based DIA, and 2.29 (95% CI, 1.17-4.48; *P* = .0134) for Aiforia-based DIA. Haralick entropy also demonstrated robust prognostic value, with HALO-based entropy showing an HR of 2.67 (95% CI, 1.36-5.26, *P* = .0031) and Aiforia-based entropy an HR of 2.71 (95% CI, 1.37-5.37, *P* = .0028), demonstrating comparable prognostic associations.

**Figure 3:**
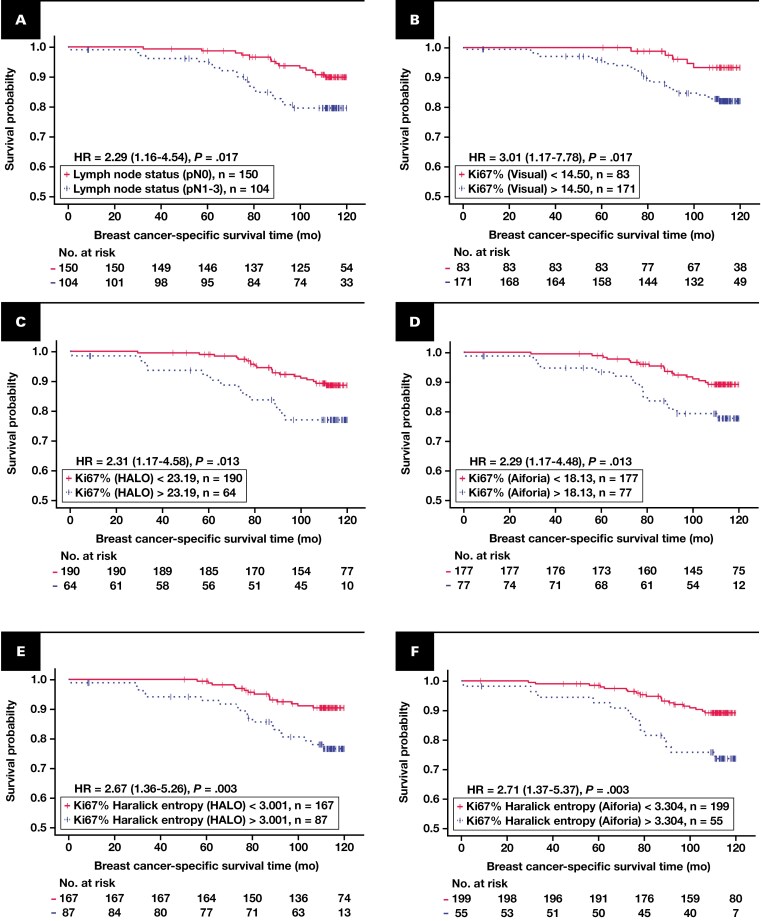
Kaplan-Meier plots for breast cancer–specific survival (BCSS) based on the independent prognostic indicators. **A**, BCSS probability stratified by pathologic lymph node status (pN0 vs pN1-3). **B**, Percentage of Ki67-positive cells in tumor tissue by pathologist report (Ki67% [Visual]). **C**, Percentage of Ki67-positive cells in tumor tissue by HALO DIA (Ki67% [HALO]). **D**, Percentage of Ki67-positive cells in tumor tissue by Aiforia DIA (Ki67% [Aiforia]). **E**, Haralick texture entropy of local ratios of Ki67-positive cells by HALO DIA (Ki67 Haralick entropy [HALO]). **F**, Haralick texture entropy of local ratios of Ki67-positive cells by Aiforia DIA (Ki67 Haralick entropy [Aiforia]). Patients were divided into 2 groups for each indicator based on optimal cutoff values determined using the Cutoff Finder.^28^ Blue dashed curves represent patients with indicator values above the cutoff, while pink solid curves represent those with values below the cutoff. Censored events, indicating patients lost to follow-up or without events by the study’s end, are marked with vertical tick marks. Statistical differences between groups were assessed using the log-rank test, and hazard ratios with 95% CIs are displayed within each plot. The number of patients at risk at various time points is shown in the table below each plot. DIA, digital image analysis; HR, hazard ratio.

Analyses across tumor subsamples and simulated core biopsy specimens reproduced these findings. For example, Ki67% in the southeast quadrant was significantly associated with worse BCSS (HR = 3.91; 95% CI, 1.19-12.78; *P* = .0150), and Haralick entropy in the right half also was a significant predictor of worse BCSS (HR = 2.27; 95% CI, 1.15-4.47; *P* = .0146). Simulated biopsy specimens further validated entropy’s prognostic utility, with biopsy 2 showing an HR of 3.10 (95% CI, 1.28-7.48; *P* = .0081). The BCSS probability plots for indicators that provided an independent prognostic impact are presented in Figure 3, while Ki67% and Haralick entropy indicators across tumor subsamples and biopsy samples are presented in Figures S2 and S3, respectively.

### Multivariable Cox regression analysis

Multivariable Cox regression analyses were performed to assess the independent prognostic value of clinicopathologic, Ki67%, and Haralick entropy metrics, structured into various subsets, as shown in Table 3. Model 1, which included clinicopathologic variables and visual Ki67% from pathology reports, identified lymph node status (HR = 2.25; 95% CI, 1.13-4.45; *P* = .0203) and visual Ki67% (HR = 2.95; 95% CI, 1.14-7.62; *P* = .0255) as independent predictors of worse BCSS (LR = 10.56, *P* = .0051; C-index = 0.671; 95% CI, 0.623-0.726).

**Table 3:** Multivariable Cox Regression Analyses of Prognostic Factors Associated With Breast Cancer-Specific Survival

Multivariable Cox regression analysis				
Indicator	Hazard ratio	*P* value	95% CI	χ^2^
**Model 1: Clinicopathologic and visual Ki67% indicators by pathology report**				
LR: 10.56, *P* = .005, mean Harrell C-index: 0.6719 (95% CI, 0.6232-0.7264)				
Lymph node status (pN0 vs pN1-3)	2.25	.02	1.13-4.45	5.38
Ki67% (Visual)	2.95	.03	1.14-7.62	4.99
**Model 2: Clinicopathologic and Ki67% indicator by HALO DIA**				
LR: 11.18, *P* = .004, mean Harrell C-index: 0.6871 (95% CI, 0.6348-0.7381)				
Lymph node status (pN0 vs pN1-3)	2.30	.02	1.16-4.55	5.68
Ki67% (HALO)	2.32	.02	1.17-4.60	5.83
**Model 3: Clinicopathologic and Ki67% indicator by Aiforia DIA**				
LR: 11.35, *P* = .003, mean Harrell C-index: 0.6878 (95% CI, 0.6412-0.7344)				
Lymph node status (pN0 vs pN1-3)	2.30	.02	1.16-4.55	5.68
Ki67% (Aiforia)	2.29	.02	1.17-4.60	5.82
**Model 4: Clinicopathologic, Ki67%, and Haralick ITH indicators by HALO DIA**				
LR: 13.67, *P* = .001, mean Harrell C-index: 0.7091 (95% CI, 0.6681-0.7574)				
Lymph node status (pN0 vs pN1-3)	2.26	.02	1.14-4.47	5.46
Ki67 Haralick entropy (HALO)	2.64	.005	1.34-5.20	7.90
**Model 5: Clinicopathologic, Ki67%, and Haralick ITH indicators by Aiforia DIA**				
LR: 13.50, *P* = .001, mean Harrell C-index: 0.7011 (95% CI, 0.6552-0.7480)				
Lymph node status (pN0 vs pN1-3)	2.33	.02	1.18-4.62	5.90
Ki67 Haralick entropy (Aiforia)	2.76	.004	1.40-5.47	8.50
**Model 6: Clinicopathologic and Ki67% indicator in the left region**				
LR: 10.31, *P* = .006, mean Harrell C-index: 0.5876 (95% CI, 0.5334-0.6532)				
Lymph node status (pN0 vs pN1-3)	2.25	.02	1.14-4.46	5.42
Ki67% (HALO, left half)	2.11	.03	1.08-4.14	4.71
**Model 7: Clinicopathologic and Ki67% indicator in the right region**				
LR: 10.02, *P* = .002, mean Harrell C-index: 0.6483 (95% CI, 0.5896-0.7001)				
Lymph node status (pN0 vs pN1-3)	2.35	.014	1.19-4.65	6.01
Ki67% (HALO, right half)	4.31	.045	1.03-18.01	4.02
**Model 8: Clinicopathologic and Ki67% indicator in the southwest region**				
LR: 10.16, *P* = .002, mean Harrell C-index: 0.5957 (95% CI, 0.5395-0.6519)				
Lymph node status (pN0 vs pN1-3)	2.40	.013	1.20-4.72	6.17
Ki67% (HALO, southwest quadrant)	2.47	.009	1.25-4.86	6.82
**Model 9: Clinicopathologic and Ki67% indicator in the southeast region**				
LR: 10.56, *P* = .001, mean Harrell C-index: 0.5618 (95% CI, 0.5041-0.6221)				
Lymph node status (pN0 vs pN1-3)	2.36	.014	1.19-4.68	6.09
Ki67% (HALO, southeast quadrant)	4.05	.021	1.24-13.24	5.34
**Model 10: Clinicopathologic and Ki67% indicator in the northwest region**				
LR: 10.23, *P* = .001, mean Harrell C-index: 0.5751 (95% CI, 0.5159-0.6326)				
Lymph node status (pN0 vs pN1-3)	2.37	.013	1.20-4.70	6.12
Ki67% (HALO, northwest quadrant)	2.66	.009	1.27-5.57	6.74
**Model 11: Clinicopathologic and Ki67% indicator in the northeast region**				
LR: 10.32, *P* = .006, mean Harrell C-index: 0.5779 (95% CI, 0.5166-0.6336)				
Lymph node status (pN0 vs pN1-3)	2.23	.021	1.13-4.42	5.30
Ki67% (HALO, northeast quadrant)	2.09	.032	1.06-4.10	4.58
**Model 12: Clinicopathologic and Ki67% indicator in the simulated biopsy 1**				
LR: 10.32, *P* = .006, mean Harrell C-index: 0.5976 (95% CI, 0.5401-0.6560)				
Lymph node status (pN0 vs pN1-3)	2.79	.019	1.15-4.49	5.51
Ki67% (HALO, biopsy 1)	2.13	.029	1.08-4.20	4.80
**Model 13: Clinicopathologic and Ki67% indicator in the simulated biopsy 2**				
LR: 10.04, *P* = .007, mean Harrell C-index: 0.5843 (95% CI, 0.5248-0.6477)				
Lymph node status (pN0 vs pN1-3)	2.29	.018	1.16-4.53	5.65
Ki67% (HALO, biopsy 2)	2.05	.037	1.05-4.01	4.35
**Model 14: Clinicopathologic, Ki67%, and Haralick ITH indicators in the left region**				
LR: 12.10, *P* = .004, mean Harrell C-index: 0.6729 (95% CI, 0.6259-0.7230)				
Lymph node status (pN0 vs pN1-3)	2.21	.023	1.12-4.38	5.17
Ki67 Haralick entropy (HALO, left half)	2.22	.020	1.13-4.36	5.38
**Model 15: Clinicopathologic, Ki67%, and Haralick ITH indicators in the right region**				
LR: 12.49, *P* = .003, mean Harrell C-index: 0.6881 (95% CI, 0.6372-0.7336)				
Lymph node status (pN0 vs pN1-3)	2.36	.014	1.19-4.67	6.04
Ki67 Haralick entropy (HALO, right half)	2.35	.014	1.19-4.62	6.08
**Model 16: Clinicopathologic, Ki67%, and Haralick ITH indicators in the southwest region**				
LR: 12.01, *P* = .003, mean Harrell C-index: 0.6829 (95% CI, 0.6318-0.7296)				
Lymph node status (pN0 vs pN1-3)	2.35	.014	1.19-4.65	6.00
Ki67 Haralick entropy (HALO, southwest quadrant)	2.67	.008	1.30-5.49	7.18
**Model 17: Clinicopathologic, Ki67%, and Haralick ITH indicators in the southeast region**				
LR: 12.54, *P* = .003, mean Harrell C-index: 0.6995 (95% CI, 0.6584-0.7485)				
Lymph node status (pN0 vs pN1-3)	2.20	.024	1.11-4.36	5.09
Ki67 Haralick entropy (HALO, southeast quadrant)	2.29	.017	1.16-4.51	5.74
**Model 18: Clinicopathologic, Ki67%, and Haralick ITH indicators in the northwest region**				
LR: 13.06, *P* = .001, mean Harrell C-index: 0.6470 (95% CI, 0.5869-0.7054)				
Lymph node status (pN0 vs pN1-3)	2.26	.019	1.14-4.48	5.49
Ki67 Haralick entropy (HALO, northwest quadrant)	2.73	.005	1.35-5.51	7.81
**Model 19: Clinicopathologic, Ki67%, and Haralick ITH indicators in the northeast region**				
LR: 12.56, *P* = .002, mean Harrell C-index: 0.6560 (95% CI, 0.6080-0.7020)				
Lymph node status (pN0 vs pN1-3)	2.35	.014	1.19-4.67	6.02
Ki67 Haralick entropy (HALO, northeast quadrant)	2.51	.008	1.28-4.92	7.13
**Model 20: Clinicopathologic, Ki67%, and Haralick ITH indicators in the simulated biopsy 1**				
LR: 12.55, *P* = .003, mean Harrell C-index: 0.6595 (95% CI, 0.6077-0.7089)				
Lymph node status (pN0 vs pN1-3)	2.33	.016	1.17-4.61	5.86
Ki67 Haralick entropy (HALO, biopsy 1)	2.29	.017	1.16-4.54	5.66
**Model 21: Clinicopathologic, Ki67%, and Haralick ITH indicators in the simulated biopsy 2**				
LR: 12.18, *P* = .004, mean Harrell C-index: 0.6118 (95% CI, 0.5610-0.6667)				
Lymph node status (pN0 vs pN1-3)	2.38	.013	1.20-4.71	6.14
Ki67 Haralick entropy (HALO, biopsy 2)	3.31	.008	1.37-8.04	7.04

Abbreviations: DIA, digital image analysis; ITH, intratumoral heterogeneity; C-index, concordance index; LR, likelihood ratio.

Models 2 and 3, incorporating DIA-derived Ki67% metrics from HALO and Aiforia, replaced visual Ki67% and demonstrated improved prognostic power with LRs of 11.18 (*P* = .0037, HALO) and 11.35 (*P* = .0034, Aiforia) and C-indices of 0.687 (95% CI, 0.635-0.738) and 0.688 (95% CI, 0.641-0.734), respectively. Ki67% remained an independent predictor of worse BCSS in both models (HALO: HR = 2.32; 95% CI, 1.17-4.60; *P* = .0158; Aiforia: HR = 2.29; 95% CI, 1.17-4.60; *P* = .0158). Compared to models 2 and 3 (which included lymph node status and Ki67% only), the addition of Haralick entropy in models 4 and 5 further enhanced prognostic performance, achieving the highest LR values (HALO: 13.67, *P* = .0011; Aiforia: 13.50, *P* = .0012) and C-indices (HALO: 0.709; 95% CI, 0.668-0.757; Aiforia: 0.701; 95% CI, 0.655-0.748), with entropy identified as an independent predictor (HALO: HR = 2.64; 95% CI, 1.34-5.20; *P* = .0049; Aiforia: HR = 2.76; 95% CI, 1.40-5.47; *P* = .0035) alongside lymph node status.

Analyses of HALO-derived Ki67% demonstrated stable prognostic significance across subsampled tumor regions, although the LRs were generally lower compared to full tumor models. For example, model 7 (right half of tumor tissue) had an HR of 4.31 (95% CI, 1.03-18.01; *P* = .045) with an LR of 10.02 (*P* = .0024) and a C-index of 0.648 (95% CI, 0.59-0.7), while model 9 (southeast quadrant) had an HR of 4.05 (95% CI, 1.24-13.24; *P* = .021) with an LR of 10.56 (*P* = .0011) and a C-index of 0.562 (95% CI, 0.504-0.622).

By adding the indicators of ITH to the data set for the subsampled tumor regions and simulated biopsy specimens, Haralick entropy confirmed its prognostic utility. Models 14 to 21 showed consistent prognostic significance, with entropy from subsampled regions (eg, model 15 [right half]: HR: 2.35; 95% CI, 1.19-4.62; *P* = .014; LR: 12.49) and simulated biopsy specimens (eg, model 20 [biopsy 1]: HR = 2.29; 95% CI, 1.16-4.54; *P* = .017; LR = 12.55) remaining independent predictors of worse BCSS. Notably, model 21 (biopsy 2) achieved the highest HR of 3.31 (95% CI, 1.37-8.04; *P* = .008), with an LR of 12.18, underscoring its strong prognostic potential.

## DISCUSSION

This study demonstrates the reproducibility and clinical relevance of Haralick texture entropy for assessing Ki67 ITH in ER+/HER2– BC. Using 2 independent DIA platforms, HALO and Aiforia, with subsequent hexagonal tiling of the DIA outputs, we demonstrated the robustness of Haralick entropy as a platform-independent metric. Both HALO- and Aiforia-derived entropy metrics were strong prognostic indicators and showed superior prognostic model performance compared to conventional Ki67% estimates in predicting worse BCSS, whether evaluated by DIA or visual assessment by pathologists. Furthermore, Haralick entropy retained its prognostic value across all simulated tissue sampling conditions, including systematically subsampled tumor regions and simulated biopsy cores, highlighting its potential for clinical application.

Unlike visual Ki67% scoring, which faces reproducibility challenges and lacks standardized assessment methods,^29^ Ki67 ITH measured as Haralick entropy offers a potentially more informative and reproducible metric for the assessment of proliferative properties in BC. In this study, a blinded analysis of 2 independent DIA platforms revealed exceptional concordance in Haralick entropy values, with Spearman correlation and ICC both reaching 0.94. While automated Ki67% scoring methods may improve reproducibility compared to visual estimates, Haralick entropy demonstrated platform-independent consistency and adds complementary information by capturing spatial heterogeneity. This reproducibility mitigates concerns about DIA platform-dependent variability and encourages broader clinical adoption of spatial entropy-based metrics.

Prognostic modeling incorporating Haralick entropy, derived from HALO and Aiforia platforms, yielded highly consistent results regardless of platform- or operator-dependent variations in the WSI segmentation, cell detection, and quantification algorithms. In both data sets, Haralick entropy emerged as an independent predictor of worse BCSS, generating the most informative Cox regression models, with HRs of 2.64 (95% CI, 1.34-5.20; *P* = .0049) for HALO and 2.76 (95% CI, 1.40-5.47; *P* = .0035) for Aiforia, alongside lymph node involvement. The LR tests confirmed model consistency, with LR values of 13.67 for HALO (*P* = .0011) and 13.50 for Aiforia (*P* = .0012). Importantly, Haralick entropy also achieved higher C-index values (0.709 [95% CI, 0.668-0.757] for HALO; 0.701 [95% CI, 0.655-0.748] for Aiforia) compared to visual Ki67% (0.672; 95% CI, 0.623-0.726) and DIA-derived Ki67% (0.687-0.688; 95% CI, 0.635-0.738), underscoring the potential added value of metrics that capture both local proportion of proliferating tumor cells and regional variance of the latter. Moreover, when added to the data sets of the models along with Ki67% and lymph node status (Table 3, models 2 and 3), the entropy further improved prognostic performance (models 4 and 5), revealing its added and independent prognostic contribution. By quantifying the spatial heterogeneity, Haralick entropy represents an alternative property of the tumor proliferation pattern, linearly independent of the Ki67% level, as reported previously.^12,14,19^ To assess whether the prognostic value of Ki67% and Haralick entropy was dependent on the choice of cutoff, we additionally explored multivariable Cox models using continuous variables. These exploratory analyses confirmed the adverse prognostic association of higher entropy values; for example, higher HALO-derived entropy was significantly associated with worse BCSS when included as a continuous variable in the model (HR = 1.30; 95% CI, 1.01-1.67; *P* = .0385; data not shown). However, effect sizes were weaker and model discrimination was lower compared to dichotomized analyses. Nevertheless, these findings suggest that the prognostic value of entropy is not solely dependent on dichotomization, supporting its robustness across different modeling approaches.

These findings align with previous studies highlighting the prognostic power of spatial heterogeneity metrics, including Ki67 bimodality,^12,19^ Shannon entropy of Ki67%,^10^ and Haralick entropy for other biomarkers like PR^12^ and HER2.^13^ Spatial heterogeneity of mesenchymal-epithelial transition protein has also been shown to outperform average marker expression in predicting survival outcomes in colorectal cancer.^24^ Altogether, this evidence reinforces the broader adoption of spatial heterogeneity metrics for improving prognostic modeling.

Our experiments on Haralick entropy under varied subsampling conditions revealed strong reproducibility of both the entropy metric and its associated prognostic models, even on a single simulated biopsy core. Using hexagonal tiling, we systematically divided WSIs of surgically excised tumor samples into smaller subregions to determine whether entropy metrics consistently reproduced their prognostic impact across various sampling sizes—a critical factor in clinical settings where minimum tissue sampling requirements need to be established. Because the tissue sections are randomly oriented when mounted, we deliberately applied systematic, algorithm-driven approaches that were unbiased with respect to slide orientation or visually defined regions, thereby simulating realistic spatial sampling variability. This design aimed to assess the robustness of the spatial metrics under semi-random but reproducible conditions. Although not biologically predefined, our spatial subsampling strategies—including half-section, quadrant-based, and simulated biopsy-level divisions—introduced orientation-agnostic randomness that effectively mimics uniform multiregional sampling. As such, they provide a practical and reproducible alternative to subjectively defined tissue compartments, particularly for spatial biomarkers, where regional heterogeneity influences interpretation. Both Ki67% and Haralick entropy demonstrated remarkable stability, with HRs and model performance metrics comparable to those obtained from full-section WSI analyses Table 3. Importantly, our experiments involved simulated subsampling within the same WSI, rather than direct comparisons of Ki67% between biopsy and surgical excision samples, as previously reported by Acs et al^30^; in fact, our data indirectly support their notion that preanalytical factors, such as delayed fixation in surgical excision samples, which may cause persistent cell division in hypoxic conditions and/or epitope degradation, can decrease the Ki67% values. In contrast, our data are based on the same WSI and therefore rule out any impact of the preanalytical factors.

Several limitations of our study must be acknowledged. First, the retrospective design lacked detailed therapy data, which may have influenced the observed patient outcomes. Future studies with comprehensive data collection and long-term follow-up are needed to validate our findings. Second, sociodemographic factors affecting follow-up and access to care were not analyzed, representing a potential area for future investigation, as these factors may impact treatment outcomes and survival. Third, while Haralick entropy demonstrated strong reproducibility across 2 DIA platforms, the generalizability of this metric requires further validation through studies using multicentric data sets, diverse DIA platforms, and larger, more diverse patient cohorts. Fourth, the cutoff values used for survival analysis were derived within the same cohort, which may introduce a risk of overfitting. Although the identified thresholds align with previously reported ranges, their prognostic relevance should be interpreted with caution and validated in independent datasets. Finally, the limited number of events (n = 34) in this study may also have reduced the statistical power of the models, underscoring the need for future analyses to confirm the clinical value of Ki67 Haralick entropy in the context of conventional clinical, pathological, and molecular features of BC.

In conclusion, Ki67 Haralick texture entropy offers a reproducible DIA-based indicator that provides independent prognostic value in patients with ER+/HER2– BC and demonstrates improved prognostic model performance compared to conventional Ki67% metrics. It may therefore serve as a robust and explicit indicator to supplement multimodal prognostic models for broader clinical adoption.

## Supplementary Material

aqaf081_suppl_Supplementary_Table_S1

aqaf081_suppl_Supplementary_Table_S2

aqaf081_suppl_Supplementary_Figure_S1

aqaf081_suppl_Supplementary_Figure_S2

aqaf081_suppl_Supplementary_Figure_S3

## Data Availability

The data underlying this article will be shared at reasonable request to the corresponding author.
