# Supplementary material for: Reproducibility of Ki67 Haralick entropy as a prognostic marker in estrogen receptor–positive HER2-negative breast cancer
Source: Am J Clin Pathol. 2025 Aug 9;164(4):567–80. doi: 10.1093/ajcp/aqaf081 (PMC12495521; doi:10.1093/ajcp/aqaf081)
Supplement: aqaf081_suppl_Supplementary_Table_S1 [file aqaf081_suppl_supplementary_table_s1.pdf]

**SUPPLEMENTARY TABLE 1** Clinicopathological characteristics of patients stratified by 10-year breast cancer-specific survival status

| Clinicopathological variables | All patients (n = 254) | No breast cancer-specific death (n = 220) | Breast cancer-specific death (n = 34) | *p*-value[a] |
|---|---|---|---|---|
| **Age at the time of surgery, years** | | | | 0.0517 |
| Median | 62 | 61 | 66 | |
| Range | 36–88 | 36–87 | 42–88 | |
| **Sex assigned at birth, n (%)** | | | | – |
| Female | 254 (100%) | 220 (100%) | 34 (100%) | |
| **Follow-up of breast cancer-specific survival, months** | | | | < 0.0001 |
| Mean | 106.5 | 111.4 | 74.4 | |
| Median | 114.8 | 116.4 | 78.2 | |
| Range | 0.8–120 | 8.4–120 | 0.8–110.9 | |
| **Stage at diagnosis, n (%)** | | | | 0.1520 |
| I | 98 (38.6%) | 89 (40.5%) | 9 (26.5%) | |
| II | 130 (51.2%) | 111 (50.5%) | 19 (55.9%) | |
| III | 26 (10.2%) | 20 (9.1%) | 6 (17.6%) | |
| **Tumor invasion stage (pT), n (%)** | | | | 0.2566 |
| pT1 | 146 (57.5%) | 130 (59.1%) | 16 (47.1%) | |
| pT2 | 108 (42.5%) | 90 (40.9%) | 18 (52.9%) | |
| **Lymph node status (pN), n (%)** | | | | 0.1161 |
| pN0 | 150 (59.1%) | 136 (61.8%) | 14 (41.2%) | |
| pN1 | 78 (30.7%) | 63 (28.6%) | 15 (44.1%) | |
| pN2 | 19 (7.5%) | 16 (7.3%) | 3 (8.8%) | |
| pN3 | 7 (2.7%) | 5 (2.3%) | 2 (5.9%) | |
| **Histological grade (G), n (%)** | | | | 0.0643 |
| G1 | 40 (15.7%) | 35 (15.9%) | 5 (14.7%) | |
| G2 | 150 (59.1%) | 135 (61.4%) | 15 (44.1%) | |
| G3 | 64 (25.2%) | 50 (22.7%) | 14 (41.2%) | |
| **Histological type, n (%)** | | | | – |
| Invasive breast carcinoma of no special type | 254 (100%) | 220 (100%) | 34 (100%) | |
| **Surrogate intrinsic BC subtype, n (%)** | | | | 0.2965 |
| Luminal A-like BC | 122 (48.0%) | 109 (49.5%) | 13 (38.2%) | |
| Luminal B-like (HER2–) BC | 132 (52.0%) | 111 (50.5%) | 21 (61.8%) | |

[a]*p*-values were calculated using the Mann–Whitney U test for continuous variables (age), and Chi-square or Fisher's exact test for categorical variables, as appropriate. BC: breast cancer; HER2–: human epidermal growth factor receptor 2-negative.
