# Supplementary material for: Reproducibility of Ki67 Haralick entropy as a prognostic marker in estrogen receptor–positive HER2-negative breast cancer
Source: Am J Clin Pathol. 2025 Aug 9;164(4):567–80. doi: 10.1093/ajcp/aqaf081 (PMC12495521; doi:10.1093/ajcp/aqaf081)
Supplement: aqaf081_suppl_Supplementary_Table_S2 [file aqaf081_suppl_supplementary_table_s2.pdf]

SUPPLEMENTARY TABLE 1 Comparison for Ki67% and Haralick's entropy indicators by HALO digital image analysis across tumor subsamples and simulated core biopsy samples

Comparison between tumor subsamples	Spearman correlation coefficient	*p*-value	Intraclass correlation coefficient (ICC)	95% confidence interval	Concordance correlation coefficient (CCC)	95% confidence interval	Bland-Altman mean difference	Limits of agreement
**Ki67%**								
Right vs. left	0.972	< 0.0001	0.968	0.953–0.981	0.969	0.954–0.981	0.105	-7.110–7.320
Right vs. overall	0.983	< 0.0001	0.984	0.973–0.992	0.984	0.972–0.992	-0.067	-5.181–5.046
Left vs. overall	0.984	< 0.0001	0.988	0.976–0.994	0.987	0.976–0.994	-0.172	-4.732–4.387
Northeast vs. overall	0.973	< 0.0001	0.978	0.965–0.986	0.978	0.966–0.986	-0.261	-6.384–5.861
Northwest vs. overall	0.975	< 0.0001	0.980	0.967–0.987	0.979	0.965–0.988	-0.197	-6.039–5.645
Southeast vs. overall	0.971	< 0.0001	0.976	0.962–0.986	0.976	0.962–0.985	-0.023	-6.411–6.365
Southwest vs. overall	0.967	< 0.0001	0.973	0.954–0.985	0.973	0.956–0.985	-0.168	-6.890–6.555
Northeast vs. northwest	0.965	< 0.0001	0.964	0.947–0.977	0.963	0.944–0.978	-0.064	-7.931–7.803
Northeast vs. southeast	0.959	< 0.0001	0.963	0.937–0.978	0.962	0.938–0.978	-0.239	-8.191–7.714
Northeast vs. southwest	0.941	< 0.0001	0.945	0.922–0.962	0.945	0.922–0.962	-0.094	-9.764–9.577
Northwest vs. southeast	0.945	< 0.0001	0.945	0.924–0.962	0.946	0.923–0.963	-0.175	-9.736–9.387
Northwest vs. southwest	0.950	< 0.0001	0.956	0.938–0.970	0.955	0.936–0.970	-0.029	-8.659–8.600
Southeast vs. southwest	0.957	< 0.0001	0.960	0.942–0.975	0.959	0.940–0.974	0.145	-8.117–8.407
Biopsy 1 vs. biopsy 2	0.967	< 0.0001	0.976	0.965–0.984	0.975	0.964–0.984	-0.187	-6.593–6.219
Biopsy 1 vs. overall	0.977	< 0.0001	0.983	0.971–0.990	0.982	0.971–0.990	-0.359	-5.735–5.016
Biopsy 2 vs. overall	0.969	< 0.0001	0.980	0.968–0.988	0.980	0.967–0.988	-0.172	-6.010–5.665
**Ki67 Haralick's entropy**								
Right vs. left	0.945	< 0.0001	0.947	0.933–0.958	0.947	0.934–0.959	-0.038	-0.914–0.839
Right vs. overall	0.983	< 0.0001	0.983	0.978–0.988	0.983	0.978–0.988	-0.080	-0.556–0.396
Left vs. overall	0.985	< 0.0001	0.985	0.981–0.989	0.985	0.981–0.989	-0.042	-0.502–0.418
Northeast vs. overall	0.945	< 0.0001	0.942	0.901–0.965	0.942	0.904–0.965	-0.163	-1.044–0.719
Northwest vs. overall	0.956	< 0.0001	0.952	0.939–0.964	0.952	0.938–0.963	-0.154	-0.940–0.632
Southeast vs. overall	0.961	< 0.0001	0.953	0.941–0.964	0.952	0.940–0.963	-0.171	-0.938–0.596
Southwest vs. overall	0.949	< 0.0001	0.941	0.920–0.957	0.942	0.922–0.958	-0.175	-1.025–0.675
Northeast vs. northwest	0.916	< 0.0001	0.920	0.891–0.941	0.919	0.889–0.942	-0.009	-1.081–1.063
Northeast vs. southeast	0.900	< 0.0001	0.899	0.856–0.932	0.898	0.853–0.931	0.009	-1.204–1.222
Northeast vs. southwest	0.882	< 0.0001	0.879	0.840–0.909	0.879	0.838–0.912	0.013	-1.283–1.309
Northwest vs. southeast	0.892	< 0.0001	0.891	0.862–0.914	0.890	0.863–0.914	0.017	-1.235–1.270
Northwest vs. southwest	0.889	< 0.0001	0.887	0.856–0.915	0.887	0.855–0.914	0.021	-1.229–1.272
Southeast vs. southwest	0.900	< 0.0001	0.899	0.866–0.925	0.898	0.866–0.926	0.004	-1.182–1.190
Biopsy 1 vs. biopsy 2	0.895	< 0.0001	0.898	0.860–0.928	0.898	0.861–0.927	0.021	-1.169–1.210
Biopsy 1 vs. overall	0.960	< 0.0001	0.949	0.936–0.960	0.949	0.936–0.960	-0.239	-0.972–0.494
Biopsy 2 vs. overall	0.931	< 0.0001	0.916	0.875–0.946	0.915	0.870–0.947	-0.260	-1.251–0.731

"Overall" refers to measurements taken across the whole tumor tissue sample.
