# Supplementary material for: Reproducibility of Ki67 Haralick entropy as a prognostic marker in estrogen receptor–positive HER2-negative breast cancer
Source: Am J Clin Pathol. 2025 Aug 9;164(4):567–80. doi: 10.1093/ajcp/aqaf081 (PMC12495521; doi:10.1093/ajcp/aqaf081)
Supplement: aqaf081_suppl_Supplementary_Figure_S1 [file aqaf081_suppl_supplementary_figure_s1.pdf]

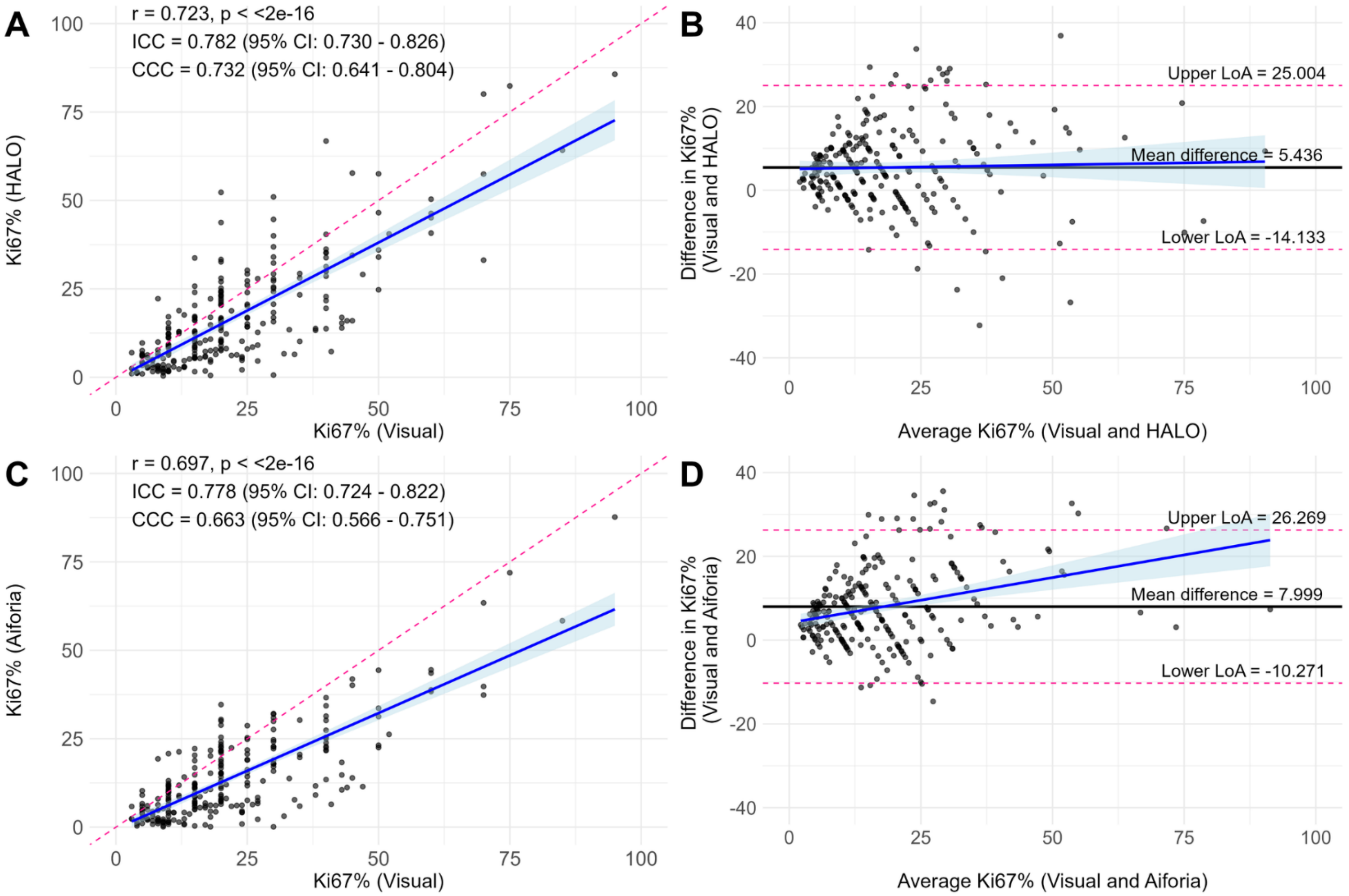

SUPPLEMENTARY FIGURE 1 Comparisons of Ki67% estimates from visual assessment ("Visual") and digital image analysis (DIA) platforms HALO and Aiforia. **A, C,** Scatter plots for pairwise comparisons: **A**, Visual vs. HALO; **C**, Visual vs. Aiforia. The dashed pink line represents the identity line (1:1), and the blue solid line indicates the linear regression fit with a 95% confidence interval (CI) shown as light blue shading. Each plot includes Spearman's correlation coefficient (*r*) with *p*-value, intraclass correlation coefficient (ICC) with 95% CI, and concordance correlation coefficient (CCC) with 95% CI to quantify agreement. **B, D,** Bland–Altman plots for the same comparisons: **B**, Visual vs. HALO; **D**, Visual vs. Aiforia. The solid black line represents the mean difference, while the dashed pink lines indicate the limits of agreement (LoA), defined as ±1.96 standard deviations from the mean difference. The blue solid line shows the linear regression of differences, with a 95% CI represented by light blue shading.
