# Supplementary material for: Reproducibility of Ki67 Haralick entropy as a prognostic marker in estrogen receptor–positive HER2-negative breast cancer
Source: Am J Clin Pathol. 2025 Aug 9;164(4):567–80. doi: 10.1093/ajcp/aqaf081 (PMC12495521; doi:10.1093/ajcp/aqaf081)
Supplement: aqaf081_suppl_Supplementary_Figure_S3 [file aqaf081_suppl_supplementary_figure_s3.pdf]

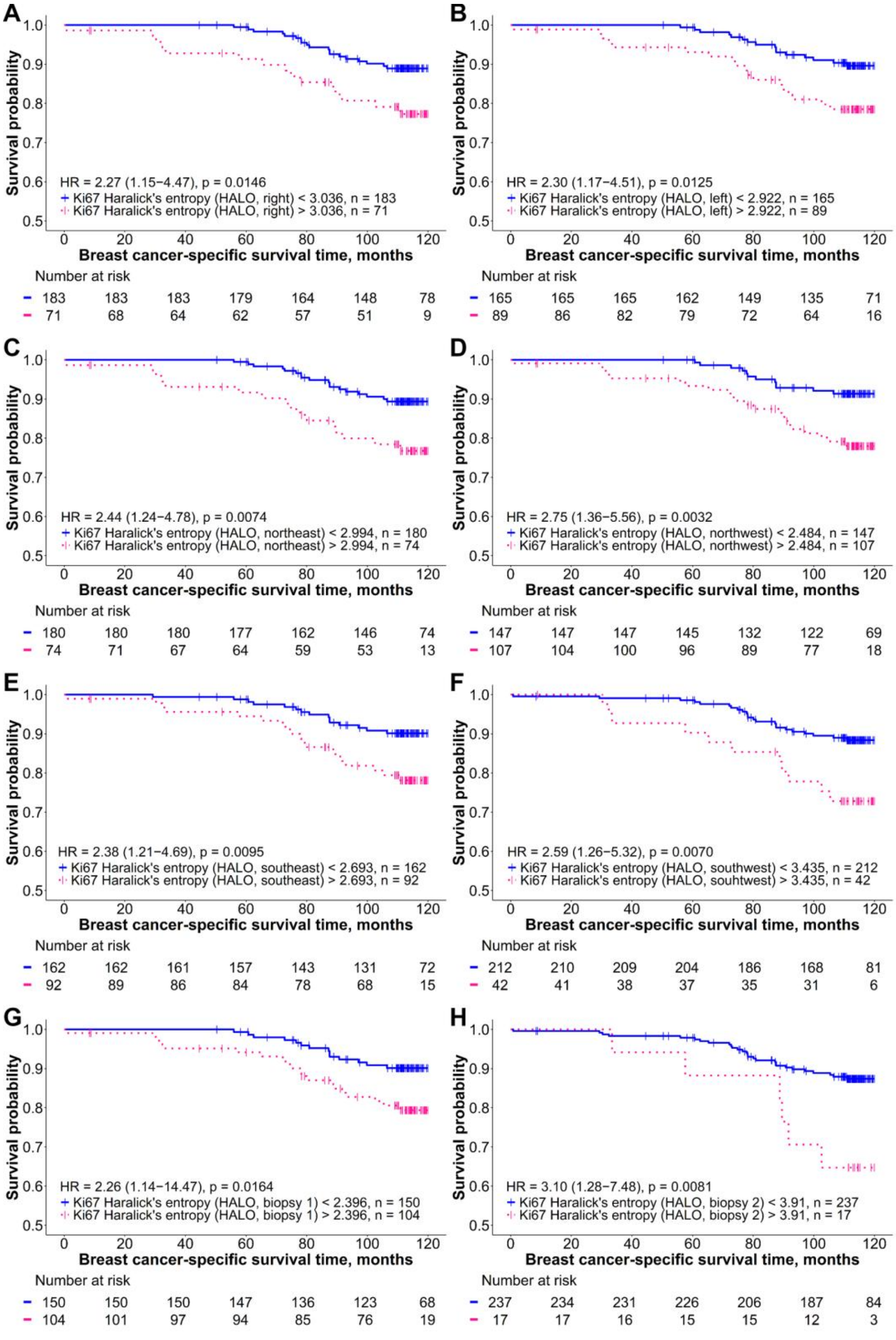

SUPPLEMENTARY FIGURE 3 Kaplan–Meier plots for breast cancer-specific survival (BCSS) based on the Ki67 Haralick's entropy across tumor subsamples and simulated core biopsies. **A,** BCSS probability stratified by Ki67 Haralick's entropy in the right tumor tissue region. **B,** BCSS probability stratified by Ki67 Haralick's entropy in the left tumor tissue region. **C,** BCSS probability stratified by Ki67 Haralick's entropy in the northeast tumor tissue region. **D,** BCSS probability stratified by Ki67 Haralick's entropy in the northwest tumor tissue region. **E,** BCSS probability stratified by Ki67 Haralick's entropy in the southeast tumor tissue region. **F,** BCSS probability stratified by Ki67 Haralick's entropy in the southwest tumor tissue region. **G,** BCSS probability stratified by Ki67 Haralick's entropy in the biopsy 1. **H,** BCSS probability stratified by Ki67 Haralick's entropy in the biopsy 2. Patients were divided into two groups for each indicator based on optimal cutoff values determined using the Cutoff Finder. Blue solid curves represent patients with indicator values below the cutoff, while pink dashed curves represent those with values above the cutoff. Censored events, indicating patients lost to follow-up or without events by the study's end, are marked with vertical tick marks. Statistical differences between groups were assessed using the log-rank test, and hazard ratios with 95% confidence intervals are displayed within each plot. The number of patients at risk at various time points is shown in the table below each plot. HR: hazard ratio.
